# MIC Distributions and CLSI-Categorized Resistance in *Pseudomonas aeruginosa* from Companion Animals in Poland: Evidence of Strong Meropenem–Ceftazidime Co-Non-Susceptibility

**DOI:** 10.3390/microorganisms14020374

**Published:** 2026-02-05

**Authors:** Dawid Jańczak, Piotr Górecki, Weronika Wójtowicz, Olga Szaluś-Jordanow

**Affiliations:** 1 Department of Infectious and Invasive Diseases and Veterinary Administration, Institute of Veterinary Medicine, Faculty of Biological and Veterinary Sciences, Nicolaus Copernicus University, Lwowska 1, 87-100 Toruń, Poland; 2 Animallab Veterinary Laboratory in Warsaw, Środkowa 2/4, 03-430 Warsaw, Poland; piotrg730@gmail.com; 3 Animallab Veterinary Laboratory in Łódź, Ignacego Paderewskiego 6, 93-509 Łódź, Poland; wwojtowicz97@gmail.com; 4 Department of Small Animal Diseases with Clinic, Institute of Veterinary Medicine, Warsaw University of Life Sciences-SGGW, Nowoursynowska 159c, 02-776 Warsaw, Poland

**Keywords:** CLSI M100, antipseudomonal agents, chronic otitis externa, feline chronic rhinitis, co-non-susceptibility, multidrug-resistant phenotype, One Health, antimicrobial stewardship

## Abstract

*Pseudomonas aeruginosa* is a clinically important opportunistic pathogen in dogs and cats, frequently associated with chronic infections and increasing antimicrobial resistance. In 2024, 111 *P. aeruginosa* isolates from 77 dogs and 34 cats were analyzed. Isolates originated from the external ear canal of animals with chronic otitis externa (66/111, 59.5%) and the nasal cavity of animals with chronic rhinitis (29/111, 26.1%), wounds (7/111, 6.3%), the conjunctival sac (5/111, 4.5%), and the skin (4/111, 3.6%). MICs for ciprofloxacin, meropenem, ceftazidime, aztreonam, piperacillin, piperacillin/tazobactam, and colistin were determined using a commercial microdilution panel and interpreted with CLSI M100 breakpoints for *P. aeruginosa*. Susceptibility was highest to piperacillin and piperacillin/tazobactam (both 90.1% susceptible and 7.2% resistant). Resistance was more frequent to ciprofloxacin (26.1%), meropenem (17.1%), and ceftazidime (16.2%). Colistin resistance (MIC ≥ 4 µg/mL) was detected in 6.3% of isolates. MDR (Magiorakos definition; non-susceptibility to ≥1 agent in ≥3 antimicrobial categories) was identified in 17/111 (15.3%) isolates. Meropenem non-susceptibility was strongly associated with ceftazidime non-susceptibility (25/111, 22.5%; OR 11.21; 95% CI 4.29–29.30; phi 0.51; *p* = 2.4 × 10^−7^). These findings provide baseline phenotypic surveillance data for *P. aeruginosa* from companion animals in Poland and highlight clinically relevant co-non-susceptibility patterns involving meropenem and ceftazidime.

## 1. Introduction

*Pseudomonas aeruginosa* is a ubiquitous Gram-negative opportunistic pathogen and a major cause of chronic, difficult-to-treat infections in both human and veterinary medicine [1,2]. Antimicrobial resistance (AMR) in *P. aeruginosa* is of One Health relevance because resistant strains and/or resistance determinants can circulate at the human–animal–environment interface, making this species a useful sentinel of selection pressure exerted by critically important antimicrobial classes [3]. In companion animals, *P. aeruginosa* is most often associated with chronic inflammatory conditions, particularly canine otitis externa and complicated or persistent upper respiratory tract disease in cats [4,5]. Its intrinsic resistance, tendency to form biofilms, and ability to acquire additional resistance determinants translate into frequent treatment failure and sustained antimicrobial selection pressure in clinical settings [6]. Chronic otitis and persistent rhinitis or sinusitis is characterized by repeated veterinary consultations and recurrent antimicrobial exposure, which together favor the emergence and maintenance of multidrug-resistant phenotypes [7,8]. Consequently, robust phenotypic characterization using MIC distributions and standardized interpretation is essential for rational therapy and for generating clinically actionable surveillance data [9].

At the same time, the epidemiology of AMR is determined by antimicrobial use practices and the broader ecological context in which resistant organisms are selected and maintained [10,11]. In *P. aeruginosa*, multiple mechanisms including reduced permeability, efflux upregulation, and β-lactamase-related pathways may converge and manifest as cross-class non-susceptibility, complicating empiric choices in chronic disease [12]. Although direct zoonotic risk is generally considered low, household- and facility-level sharing of resistant strains has been documented, underscoring the importance of stewardship-oriented interpretation of veterinary AMR data [13,14].

Therefore, the aim of this study was to characterize MIC distributions and CLSI-interpreted susceptibility categories of *P. aeruginosa* isolates from dogs and cats in Poland [15]. We further sought to quantify co-non-susceptibility (intermediate + resistant) patterns across key antipseudomonal agents, to support evidence-based therapeutic decision-making and surveillance [16,17,18]. Because resistance phenotypes in *P. aeruginosa* tend to cluster across antipseudomonal classes, we specifically evaluated co-non-susceptibility structure and highlighted meropenem–ceftazidime co-non-susceptibility as a clinically and One Health–relevant pattern in this dataset.

## 2. Materials and Methods

### 2.1. Study Population Characteristics

The dataset included 111 animals, including 77 dogs (69.4%) and 34 cats (30.6%). Overall, 67/111 (60.4%) animals were male and 44/111 (39.6%) were female (dogs: 50 males/27 females; cats: 17 males/17 females). The odds of male were higher in dogs than in cats (OR 1.85, 95% CI 0.82–4.20). Age data were available for all 111 animals, and the median age was 5.0 years (interquartile range; IQR 2.6–8.0) ranging from 4 months to 15 years.

### 2.2. Specimen Collection

The 111 *Pseudomonas aeruginosa* isolates originated from a retrospective diagnostic cohort of clinical specimens submitted in 2024 by external veterinary clinics to a commercial laboratory (Animallab Veterinary Laboratory, Warsaw, Poland). Clinical specimens included swabs from the external ear canal (66/111; 59.5%), nasal cavity (29/111; 26.1%), wounds (7/111; 6.3%), conjunctival sac (5/111; 4.5%), and skin (4/111; 3.6%). Ear canal specimens were obtained from patients with chronic otitis externa, whereas nasal swabs were collected from animals with chronic rhinitis. In this cohort, ear canal isolates were nearly exclusively from dogs (65/66), while nasal isolates were exclusively from cats (29/29), reflecting the diagnostic case mix of submissions. All bacteriological swabs were collected for routine clinical purposes and submitted as part of standard diagnostic work-up, and only anonymized metadata were used for analysis.

### 2.3. Bacterial Culture and Antimicrobial Susceptibility Testing

Swabs were inoculated onto 5% defibrinated sheep blood agar and MacConkey agar plates (GRASO Biotech, Starogard Gdański, Poland). The plates were incubated under aerobic conditions at 35 ± 2 °C for 24, 48, and 72 h. Bacterial identification was performed using the MALDI Biotyper^®^ system, with spectra acquisition and analysis performed with the manufacturer’s software MBT Compass version 4.1 (Bruker Daltonics GmbH & Co. KG, Bremen, Germany).

Antimicrobial susceptibility was evaluated using the commercial MIC panel NEF (DIAGNOSTICS s.r.o., Galanta, Slovakia) including the following antimicrobials: ampicillin/sulbactam, ciprofloxacin, meropenem, ceftazidime, aztreonam, piperacillin, piperacillin/tazobactam, colistin, amikacin, gentamicin, tigecycline and sulfamethoxazole. MIC results were interpreted according to the Clinical and Laboratory Standards Institute (CLSI) criteria [15]. The MIC panel wells for ampicillin/sulbactam, tigecycline, and sulfamethoxazole were excluded from analysis because *Pseudomonas aeruginosa* is intrinsically non-susceptible to these agents, and/or because clinical breakpoints or recommended testing procedures are not established for *Pseudomonas* spp. [16,17,18]. Breakpoints for antipseudomonal agents are provided in Table S1. Multidrug resistance (MDR) was defined using the international consensus definition as non-susceptibility (CLSI I + R) to at least one agent in ≥3 antimicrobial categories. For MDR classification in this dataset, categories were represented as follows: fluoroquinolones (ciprofloxacin), carbapenems (meropenem), extended-spectrum cephalosporins (ceftazidime), antipseudomonal penicillins/β-lactamase inhibitor combinations (piperacillin and/or piperacillin/tazobactam; counted as one category due to full concordance), and polymyxins (colistin; no intermediate category). Aztreonam was excluded from MDR classification because the panel did not include dilutions at or above the CLSI resistant threshold (≥32 µg/mL), precluding estimation of resistance prevalence Amikacin was not used for MDR classification because CLSI breakpoints for *P. aeruginosa* are urine-only, and gentamicin was excluded due to the lack of CLSI interpretive breakpoints for *P. aeruginosa*.

### 2.4. Statistical Data Analysis

MIC values (µg/mL) were summarized as minimum–maximum, MIC50 (median), and MIC90 (90th percentile). Piperacillin and piperacillin/tazobactam were treated as a single beta-lactam class in multivariable summaries because their categories were fully concordant in this dataset. Between-group comparisons of categorical outcomes were assessed using Fisher’s exact test. Co-non-susceptibility between pairs of agents was quantified using 2 × 2 contingency tables, Fisher’s exact test, odds ratios (OR), and the phi coefficient as an effect size; non-susceptibility was defined as CLSI intermediate or resistant (I + R). Exploratory risk factor analyses were performed with binary logistic regression models (outcome: non-susceptible vs. susceptible), including age (years, continuous) and sex as predictors; to reduce confounding from the strong association between host species and sampling site, regression analyses were run within the two dominant clinical strata (dog ear isolates; cat nose isolates). Statistical significance was set at *p* < 0.05; for multiple co-non-susceptibility tests, false discovery rate control (Benjamini–Hochberg) was applied.

## 3. Results

A total of 111 *Pseudomonas aeruginosa* isolates were analyzed (dogs: 77/111, 69.4%; cats: 34/111, 30.6%). Specimens originated primarily from the ear canal (66/111, 59.5%) and nasal cavity (29/111, 26.1%), with fewer isolates from wounds (7/111, 6.3%), conjunctival sac (5/111, 4.5%), and skin (4/111, 3.6%).

**Figure 1:**
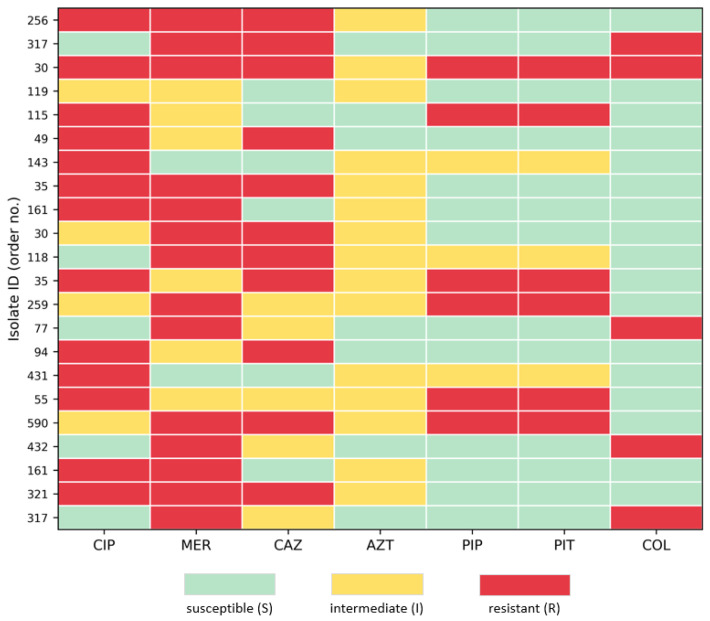
Heatmap of antimicrobial susceptibility categories for MDR *Pseudomonas aeruginosa* isolates (N = 22), defined as non-susceptibility (I + R) to at least one agent in ≥3 antimicrobial categories (CLSI M100). Only MDR isolates are shown to improve readability and to visualize resistance clustering among the most phenotypically constrained profiles; aztreonam is displayed as S/I within the tested range because the panel did not include the CLSI resistant dilution range. Each row represents a single isolate, and each column represents an antimicrobial agent (CIP, MER, CAZ, AZT, PIP, PIT, COL). Color coding indicates susceptibility categories: susceptible (S), intermediate (I), and resistant (R).

Using CLSI *P. aeruginosa* breakpoints, susceptibility was highest for antipseudomonal penicillins (PIP/PIT: 90.1% S; 7.2% R) and lower for ciprofloxacin (68.5% S; 26.1% R) and meropenem (62.2% S; 17.1% R). Ceftazidime resistance was observed in 16.2% of isolates. For colistin, 6.3% of isolates were categorized as resistant (R ≥ 4 µg/mL), while the remaining were intermediate. For aztreonam, resistance could not be directly assessed because the MIC panel did not include the CLSI-resistant range (≥32 µg/mL); isolates were classified as susceptible or intermediate within the tested range. Accordingly, aztreonam results are reported descriptively (S/I within the tested range) and were not used to derive MDR prevalence or clustering conclusions [Table 1,Figure 1].

Using the international consensus definition (non-susceptibility to ≥1 agent in ≥3 antimicrobial categories), MDR was identified in 17/111 isolates (15.3%). Co-non-susceptibility analysis demonstrated a strong association between meropenem and ceftazidime non-susceptibility (25/111, 22.5% of isolates non-susceptible to both agents; OR 11.21, 95% CI 4.29–29.30; *p* = 2.4 × 10^−7^; phi = 0.51). Complete concordance was observed between PIP and PIT categories across all isolates.

### Exploratory Risk Factor Analysis

In exploratory regression models restricted to dog ear isolates with complete covariate data (N = 57), increasing age was associated with lower odds of meropenem non-susceptibility (OR 0.80 per year; *p* = 0.021). Isolates obtained from male animals had lower odds of ceftazidime non-susceptibility than from females (male vs. female OR 0.16; *p* = 0.0049). These associations should be interpreted cautiously as exploratory findings and warrant confirmation in larger, prospectively balanced datasets.

## 4. Discussion

This report is a laboratory-based analysis describing the occurrence of *P. aeruginosa* infections in Polish companion animals, with a particular emphasis on chronic feline rhinitis and canine otitis externa. These distributions are clinically consistent with the well-established predilection of *P. aeruginosa* for chronic, inflamed, moist niches and its capacity to endure persistent local pathology and antimicrobial exposure.

Chronic otitis externa is a typical clinical setting prone to *P. aeruginosa* overgrowth, and a rod-dominant cytology pattern is a strong indication of clinical infection. Numerous reviews and diagnostic cohorts report that *P. aeruginosa* is the most clinically relevant Gram-negative pathogen in chronic otitis, particularly in cases of complicated infection, otitis media, epithelial hyperplasia, and biofilm formation that induce recurrence [4,19].

Epidemiological datasets from numerous regions support its frequent involvement in canine ear disease [20]. A recent diagnostic survey in Spain, based on 604 otitis submissions, reported significant antimicrobial resistance across otitis pathogens, underscoring the importance of culture-guided therapy in chronic conditions [21].

The etiology of feline chronic rhinitis is heterogeneous, with potential causes including viral infections, anatomic disorders, polyps, neoplasia, and foreign bodies. In a detailed assessment of cats with chronic rhinitis, *Pseudomonas* spp. represented a substantial proportion of bacterial isolates (32.1%), highlighting that *Pseudomonas* may be a significant complicating agent in chronic sinonasal disease [22]. The strong association between nasal isolates and cats in our dataset supports the clinical observation that chronic upper respiratory disease may serve as a key reservoir for *Pseudomonas* in feline medicine.

Using CLSI M100 interpretation, susceptibility in our cohort was highest for antipseudomonal penicillins (piperacillin and piperacillin/tazobactam: 90.1% susceptible; 7.2% resistant), while resistance was more frequent to ciprofloxacin (26.1% resistant), meropenem (17.1% resistant), and ceftazidime (16.2% resistant). These findings are consistent with the frequency reported in other companion-animal studies of *P. aeruginosa*, where fluoroquinolone resistance can be substantial, and beta-lactam susceptibility may vary by geography, antimicrobial stewardship norms, and intensity of early treatment. Studies focused on canine otitis typically report significantly higher resistance rates to commonly used agents. These susceptibility patterns underscore the value of culture and MIC testing in chronic or recurrent cases, particularly after prior antimicrobial exposure [4,19,21].

MIC distributions offer clinical information that surpasses categorical S/I/R reporting, particularly in chronic infections where susceptibility profiles can be altered over time as a result of repeated exposure and biofilm formation. MIC-guided selection should be interpreted in the context of the route of administration and achievable concentrations at the infection site because in otitis externa, the local drug concentrations attained by topical therapy may surpass systemic concentrations by orders of magnitude. However, chronic otitis is a high-risk scenario for the selection of resistant subpopulations, relapse, and persistence, particularly when treatment is initiated empirically or changed repeatedly without microbiological confirmation. After treatment failure, in recurrent disease, in suspected otitis media, or when rod-dominant cytology indicates Gram-negative involvement and biofilm-associated persistence, culture and susceptibility testing are most useful in these situations [4,6,19].

The variability of the primary disease drivers, such as viral damage, anatomical distortion, and inflammatory remodeling brought on by bacteria acting as secondary complicating agents rather than the only etiologic pathogens, presents a significant interpretive challenge for chronic feline rhinitis. Therefore, susceptibility results should not be considered as a standalone indication for escalation, rather than being considered in conjunction with the diagnostic work-up and clinical context. ISCAID guidance emphasizes antimicrobial selection proportional to clinical severity and the importance of diagnostic sampling, particularly in chronic respiratory disease with prior antimicrobial exposure [8]. The observed resistance proportions to ciprofloxacin and selected β-lactams are consistent with the possibility that many cases represent previously treated or recurrent disease, despite the absence of detailed treatment reports in the present dataset. Consequently, the most actionable message in both syndromes is not the promotion of specific molecules, but rather the prioritization of microbiological confirmation, the avoidance of unnecessary antimicrobial switching, and the alignment of therapeutic choices with culture/MIC results where clinically justified [4,8,9,21].

The MIC distribution patterns observed here also support a practical stratification of decision-making. Initially, the most susceptible isolates in this cohort were piperacillin and piperacillin/tazobactam. Consequently, isolates with broadly low MICs may still be treatable with standard antipseudomonal β-lactams when systemic therapy is clinically indicated. However, in chronic otitis, local therapy is the cornerstone, and the primary function of susceptibility testing is to avoid ineffective empirical regimens and to document resistance in refractory cases [4,19]. The clinical relevance of the relatively frequent non-susceptibility to ciprofloxacin is underscored by the fact that fluoroquinolones are frequently employed in small animal practice. The selection of resistant populations may be facilitated by repeated or prolonged courses, particularly in chronic disease, which could complicate future management [8,21]. Third, polymyxins are considered of critical importance in human medicine and are usually subject to stewardship restrictions. Therefore, the presence of colistin resistance, even at a low proportion, warrants attention. Colistin MIC testing in *P. aeruginosa* is technically challenging, and results can be influenced by methodological variability and heteroresistance. Therefore, colistin categorization should be interpreted cautiously and primarily as a surveillance signal in this retrospective dataset, rather than as a basis for therapeutic inference [23,24,25].

Lastly, the dataset emphasizes how critical transparency and standardized reporting are to phenotypic surveillance. For instance, the panel did not include the CLSI resistant dilution range (≥32 µg/mL), which prevented the direct assessment of aztreonam categorical resistance. Though the reporting of S/I within the tested range is still valuable, it also serves as an illustration of how panel design can limit interpretation in retrospective datasets [15]. These arguments collectively support a surveillance strategy that integrates MIC distributions, precise definitions of non-susceptibility (I + R), and clinically contextualized interpretation that prioritizes stewardship and infection control over the promotion of restricted drug use [8,9,15,23,24,25,26,27].

Using the international consensus MDR definition (non-susceptibility to ≥1 agent in ≥3 antimicrobial categories), MDR was identified in 22/111 (19.8%) isolates. Moreover, co-non-susceptibility analysis demonstrated a strong association between meropenem and ceftazidime non-susceptibility (OR 11.21; phi 0.51), consistent with convergent resistance mechanisms affecting core antipseudomonal β-lactams/carbapenems. This population distribution is in line with the documented biology of *P. aeruginosa*, which involves the spread of resistance through a combination of mechanisms (AmpC overexpression, porin reduction, efflux upregulation, and the acquisition of ESBL/MBL/carbapenemase determinants). This results in associated non-susceptibility across important classes [20,28,29].

The strong meropenem–ceftazidime association is a prime example of the marked clustering of β-lactam and carbapenem non-susceptibility, which is a central finding of this study. Phenotypically, such clustering is consistent with convergent mechanisms that jointly affect multiple antipseudomonal classes. In *P. aeruginosa*, reduced permeability and porin alterations can diminish carbapenem entry, while derepressed chromosomal AmpC activity and efflux upregulation can contribute to broader β-lactam and multidrug non-susceptibility profiles [2,12]. Horizontally acquired determinants, such as ESBLs and metallo-β-lactamases, may further complement these intrinsic and adaptive pathways, thereby enhance cross-class non-susceptibility and generate clinically challenging phenotypes [2,12,28,29].

The observed pattern is clinically meaningful because it indicates that “stepwise escalation” within β-lactams may be unreliable once a high-level resistance phenotype emerges in chronic disease. It is important to note that our analysis is phenotypic and cannot assign mechanisms. In practice, this reinforces the need to avoid repeated empirical cycling among antipseudomonal β-lactams when *P. aeruginosa* is suspected in refractory infections and to rely on MIC results for drug selection where systemic therapy is warranted [4,8,19]. Because co-non-susceptibility clustering may be an early warning indicator of selection pressure and the possible introduction or emergence of high-risk lineages, the finding is also pertinent to surveillance. Animal isolates and high-risk human sequence types have been found to overlap in recent veterinary and comparative studies. This shows that veterinary phenotypes may not always represent a completely separate reservoir for AMR [13,14]. Therefore, even without genotyping, quantifying such co-non-susceptibility structures adds value by identifying phenotypic signatures that merit prioritization for targeted sequencing and prospective follow-up [14,29].

The evaluation of colistin, aztreonam, and meropenem is particularly significant from a One Health perspective. In the EU, expert frameworks classify several of these classes as highly restricted for veterinary use. Fluoroquinolones, 3rd/4th generation cephalosporins, and polymyxins are generally restricted for animals, while carbapenems are typically not intended for veterinary use [23,24,25].

In addition, EU legislation and implementing measures provide mechanisms to reserve certain antimicrobials for human infections and limit veterinary access under specified conditions [26]. These restrictions reflect their critical importance in human medicine, also emphasized in global prioritization schemes such as the WHO list of medically important antimicrobials [27]. The objective of our study was to identify resistance in antimicrobials of critical importance to human medicine resulting from clinically significant resistance determinants present in companion animals. This work does not advocate the use of these agents in veterinary practice.

From a One Health and stewardship perspective, the most important implication of MDR phenotypes and β-lactam/carbapenem co-non-susceptibility clusters is not therapeutic expansion, but risk management. EU expert frameworks and implementing regulations prioritize restriction and reservation principles for critical antimicrobial classes, such as fluoroquinolones, 3rd/4th-generation cephalosporins, polymyxins, and carbapenems, with a particular emphasis on carbapenems [23,24,25,26]. Global prioritization schemes similarly underscore the need to mitigate selection pressure arising from non-human use and to align veterinary practice with stewardship objectives [27]. Phenotypic resistance in companion-animal *P. aeruginosa* should be interpreted as a sentinel outcome of selection pressure and as a trigger for infection control and antimicrobial-use review in this context, rather than as an indication to deploy restricted agents [23,24,25,26,27,30].

In companion-animal settings, practical stewardship actions include the following: ensuring that sampling is conducted prior to repeated antimicrobial changes in chronic disease, limiting unnecessary systemic therapy when topical/local control is feasible, using the narrowest effective option supported by MIC results, and documenting prior antimicrobial exposure to facilitate more robust risk factor analyses in future surveillance [8,9,30]. Infection control is a complementary component when resistant *P. aeruginosa* is identified. Strict hand hygiene, disinfection and cleaning of high-touch wet surfaces, cautious handling of otoscope cones and endoscopic equipment, and reducing the risk of cross-contamination during examination and sample handling are all part of this in veterinary facilities. Particularly in households with vulnerable individuals, owners should be provided with practical advice regarding the management of exudates (eye discharge, wound secretions), hygiene after handling the affected animal, and environmental cleaning of contaminated materials at the household level. Such measures are consistent with stewardship guidance emphasizing that antimicrobial resistance management requires both prudent antimicrobial use and transmission interruption strategies in clinical and domestic environments [13,30].

Although no genomic typing was performed in the present study, external reports of household or facility-level sharing of resistant *P. aeruginosa* provide contextual examples supporting One Health framing; these are not findings of the current dataset [13].

Otitis isolates from companion animals have been shown to include genotypes overlapping with those of high-risk human strains, reinforcing the idea that companion animal infections may contribute to broader AMR ecology rather than forming fully separate veterinary reservoirs [13].

From a laboratory and surveillance perspective, these findings indicate that chronic otitis externa in dogs and chronic rhinitis in cats are frequent sources of *P. aeruginosa* submissions, and that resistance to several antipseudomonal classes is present in this diagnostic population. Therefore, susceptibility testing can provide microbiological information to support case-level decision-making when clinicians judge it necessary, particularly in refractory or recurrent infections.

Lastly, the presence of MDR phenotypes and beta-lactam/carbapenem co-non-susceptibility clusters highlights the need for antimicrobial stewardship aligned with One Health priorities, with careful attention to infection control in households and veterinary facilities when resistant *P. aeruginosa* is identified [30].

Finally, to support clear reporting, a few interpretive details should be brought to light. Although direct quantification of categorical resistance was not possible due to the panel’s aztreonam MIC range not extending into the CLSI resistant range, MIC distributions and S/I categorization within the tested range are still useful for surveillance and for identifying isolates that need confirmatory testing with longer dilution ranges [15]. Secondly, the interpretation of colistin is distinct from that of many other agents due to the use of an intermediate-only category below the resistant threshold. Consequently, non-susceptibility for colistin is effectively equivalent to resistance in practice, and resistance findings should be interpreted primarily as surveillance signals under stewardship restrictions rather than as treatment. The value of reporting MIC distributions as categorical outcomes and of explicitly stating analytical definitions (non-susceptible = I + R) is supported by these details, which facilitate predictability and accessibility across laboratories and studies.

Limitations of this study are common for retrospective diagnostic datasets. This study is based on retrospective routine diagnostic submissions. Thus, the isolate distribution is primarily indicative of diagnostic case mix and submission behavior, rather than population-level prevalence. A significant structural limitation is the strong association between sampling site and host species in this cohort (primarily canine external ear canal isolates versus exclusively feline nasal isolates), which limits the ability to draw conclusions about species-effects independent of sampling location and clinical syndrome and may help to explain group differences. Furthermore, the impact of previous antimicrobial exposure, duration of disease, referral status, and repeated sampling factors that likely drive selection pressure in chronic otitis and rhinitis could not be quantified due to the lack of consistent access to detailed treatment histories and standardized clinical metadata. These factors should be addressed in future prospective designs. Finally, the present work is intentionally phenotypic and does not include genetic characterization. Thus, the specific resistance mechanisms that underlie the observed MIC patterns and potential transmission pathways are unresolved. In order to identify high-risk lineages and actionable resistance determinants in the Polish companion-animal context, future research should prioritize prospective, syndrome-stratified sampling with harmonized metadata and targeted genotyping or whole-genome sequencing of isolates exhibiting meropenem–ceftazidime co-non-susceptibility.

## 5. Conclusions

In this retrospective cohort of 111 *P. aeruginosa* isolates from companion animals in Poland (routine diagnostic submissions), MIC distributions and CLSI interpretation demonstrated substantial non-susceptibility to several antipseudomonal agents and the presence of multidrug-resistant phenotypes. MDR (non-susceptibility to ≥1 agent in ≥3 antimicrobial categories) was present in 17/111 isolates (15.3%). A key finding was the marked clustering of β-lactam non-susceptibility, particularly the strong association between meropenem and ceftazidime non-susceptibility, with 25/111 (22.5%) isolates non-susceptible to both agents. These results underscore that, in chronic clinical syndromes typically prompting repeated antimicrobial exposure, phenotypic resistance profiles in *P. aeruginosa* may rapidly limit therapeutic options and highlight that phenotypic resistance and co-non-susceptibility clustering may narrow therapeutic options in chronic presentations, supporting the epidemiological value of MIC-based surveillance and motivating prospective studies with standardized clinical metadata. Given the retrospective design and the strong host–site case mix in this dataset, prospective studies with harmonized clinical metadata and targeted genotyping are warranted to define the mechanisms and epidemiology underlying the observed co-non-susceptibility patterns.

## Figures and Tables

**Table 1:** Minimum inhibitory concentration (MIC) distributions (MIC range, MIC_50_, MIC_90_) and categorical susceptibility (susceptible/intermediate/resistant) of *Pseudomonas aeruginosa* isolates (N = 111) to the tested antimicrobial agents, interpreted according to CLSI criteria.

Antimicrobial	N	MI C Range (µg/mL)	MIC_50_	MIC_90_	Susceptible, n (%)	Intermediate, n (%)	Resistant, n (%)
Ciprofloxacin (CIP)	111	0.064–8	0.5	8	76 (68.5)	6 (5.4)	29 (26.1)
Meropenem (MER)	111	0.125–16	2	8	69 (62.2)	23 (20.7)	19 (17.1)
Ceftazidime (CAZ)	111	0.25–32	4	32	78 (70.3)	15 (13.5)	18 (16.2)
Aztreonam (AZT)	111	0.125–16	4	16	91 (82.0)	20 (18.0)	0 (0.0) ^a^
Piperacillin (PIP)	111	1–128	4	16	100 (90.1)	3 (2.7)	8 (7.2)
Piperacillin/tazobactam (PIT)	111	1–128	4	16	100 (90.1)	3 (2.7)	8 (7.2)
Colistin (COL)	111	0.25–16	2	2	— ^b^	104 (93.7)	7 (6.3)
Antimicrobial agents without CLSI categorical interpretation							
Amikacin (AMK)	111	0.5–64	4	8	— ^c^	—	—
Gentamicin (GEN)	111	0.25–32	2	4	— ^d^	—	—

Abbreviations: MIC50 = median MIC; MIC90 = 90th percentile MIC; CLSI interpretive criteria were applied using *P. aeruginosa*-specific breakpoints (CLSI M100). Breakpoints used (MIC, µg/mL): CIP S ≤ 0.5; I = 1; R ≥ 2; MER S ≤ 2; I = 4; R ≥ 8; CAZ S ≤ 8; I = 16; R ≥ 32; AZT S ≤ 8; I = 16; R ≥ 32; PIP S ≤ 16; I = 32; R ≥ 64; PIT S ≤ 16/4; I = 32/4; R ≥ 64/4; ^a^ AZT: the MIC panel in this dataset did not include dilutions ≥ 32 µg/mL (CLSI R-threshold), therefore R could not be directly assessed; values are reported as S/I within the tested range; ^b^ COL: no “Susceptible” category; report I ≤ 2 and R ≥ 4; ^c^ AMK: CLSI breakpoints for *P. aeruginosa* are urine-only; therefore, S/I/R was not assigned for non-urinary specimens; ^d^ GEN: interpretive criteria for *P. aeruginosa* are not consistently provided/recommended in current CLSI/FDA frameworks; therefore, GEN MIC distributions are reported without categorical interpretation.

## Data Availability

The original contributions presented in this study are included in the article/Supplementary Material. Further inquiries can be directed to the corresponding authors.

## Supplementary Materials

The following supporting information can be downloaded at: https://www.mdpi.com/article/10.3390/microorganisms14020374/s1. Table S1. CLSI MIC breakpoints for Pseudomonas aeruginosa (CLSI M100, 34th edition).
