# Supplementary material for: MIC Distributions and CLSI-Categorized Resistance in Pseudomonas aeruginosa from Companion Animals in Poland: Evidence of Strong Meropenem–Ceftazidime Co-Non-Susceptibility
Source: Microorganisms. 2026 Feb 5;14(2):374. doi: 10.3390/microorganisms14020374 (PMC12943731; doi:10.3390/microorganisms14020374)
Supplement: Supplementary file 1 [file microorganisms-14-00374-s001.zip › microorganisms-4130362-supplementary.pdf]

Table S1. CLSI MIC breakpoints for *Pseudomonas aeruginosa* (CLSI M100, 34th edition).

Antimicrobial agent	Susceptible (S) μg/mL	Intermediate (I) μg/mL	Resistant (R) μg/mL
Ciprofloxacin (CIP)	≤0.5	1	≥2
Meropenem (MER)	≤2	4	≥8
Ceftazidime (CAZ)	≤8	16	≥32
Aztreonam (AZT)	≤8	16	≥32
Piperacillin (PIP)	≤16	32	≥64
Piperacillin/tazobactam (PIT)	≤16/4	32/4	≥64/4
Colistin (COL)	≤2	—	≥4
Amikacin (AMK)[a]	≤16	32	≥64
Gentamicin (GEN)[b]	—	—	—

Abbreviations: [a]AMK - report only for urinary tract isolates; [b] no CLSI breakpoints for *P. aeruginosa*
